# Drivers of Green Entrepreneurial Intention: Why Does Sustainability Awareness Matter Among University Students?

**DOI:** 10.3389/fpsyg.2022.873140

**Published:** 2022-03-28

**Authors:** Hartiwi Prabowo, Ridho Bramulya Ikhsan, Yuniarty Yuniarty

**Affiliations:** Management Department, Binus Online Learning, Bina Nusantara University, Jakarta, Indonesia

**Keywords:** green entrepreneurial intention, theory of planned behavior, culture values, knowledge of cognition, contextual factors, university students

## Abstract

Entrepreneurship is an essential aspect of economic growth because of its contribution to people’s welfare through employment opportunities. Universities offer compulsory entrepreneurship subjects for students with the support of government policies. Therefore, this study aimed to determine the factors that influence the students’ intentions to become green entrepreneurs using contextual aspects as moderators. The applied theoretical model was the planned behavior (TPB) that adds cultural values and cognitive knowledge. The sample included 305 students from 10 private universities in Jakarta. The results showed that green entrepreneurial intentions are affected by perceived behavioral control (PBC), cultural values, cognition knowledge, and contextual factors. However, they lack a significant effect on attitudes toward behavior and subjective norms. Second, the contextual factors can moderate the relationship between variables and significantly affect green entrepreneurial intentions. Third, they moderate attitudes toward behavior and cognitive knowledge with green entrepreneurial intentions. Contrastingly, other factors had no effect when contextual factors moderated the relationship.

## Introduction

Small medium enterprises (SMEs) support global economic growth, including the developing countries. Based on their essential contribution to the nation, such as increasing the employment rate, the Government of Indonesia ensures that 4% of the population holds entrepreneurial professions (Safitri, 2017). This follows conventional business and increasing innovative business-oriented green and sustainability practices.

As higher education institutions, universities should produce students with business management skills based on sustainability principles. Therefore, the Indonesian government requires every university to design an entrepreneurship-based curriculum with effective simulations that adopt real business scenarios. Higher education institutions should promote entrepreneurial growth through entrepreneurship education (Marques et al., 2018; Cui et al., 2021). Several countries prioritize entrepreneurship education, though few studies focus on the impacts of this aspect. Furthermore, entrepreneurship has been introduced and promoted virtually worldwide and in the most universities (Graevenitz et al., 2010). According to Graevenitz et al. (2010), there is inadequate understanding of the impacts of entrepreneurship education, though some literature produced compelling insights.

Bosma and Kelley (2019) examined the Indonesian entrepreneurship situation and established that perceived abilities exceed opportunities. This gap should inspire new entrepreneurs, though starting a new business in Indonesia is unpopular. Specifically, the country ranked fourth in the GEM 2018–2019 Global Report on the entrepreneurial intentions of people aged 18–64, following Thailand, Korea, and Taiwan. Indonesians expressed their entrepreneurial confidence in the high-perceived opportunity rate of 54.9% and relatively high-entrepreneurial intention rate of 24%. Thailand ranked first among the seven countries within the Eastern and Southern Asian region at 30%. Indonesian universities instill entrepreneurial passion and spirit to the students through various motivating methods and strategies to become entrepreneurs, including gamification (Aries et al., 2020).

A previous study found that two-thirds of Indonesia’s adults viewed starting a business as easy as selecting a favorable career (Bosma and Kelley, 2019). Furthermore, the Government should provide sufficient infrastructure and policies to increase entrepreneurship skills as a promising career option (Minniti, 2008). Studies on entrepreneurship education have an immense gap (Graevenitz et al., 2010), with a few finding the positive impacts of entrepreneurship education programs in higher education on perceptions of the desirability and feasibility of starting a business (Fayolle et al., 2006; Marques et al., 2018; Nowiński et al., 2019; Boldureanu et al., 2020; Ndofirepi, 2020; Cui et al., 2021), while some found negative effects (Oosterbeek et al., 2010). There is no current consensus on a fitting conceptual model for analyzing entrepreneurship education impacts (Graevenitz et al., 2010). Finally, this research is expected to add new insight into the effect of TPB factors, cultural values, and knowledge of cognition on green entrepreneurship intentions with contextual factors (educational, structural, and relational support) as moderators. The results of this study are significant for universities to develop an entrepreneurship-based curriculum and government policies in motivating students to become green entrepreneurs.

## Theoretical Background

Sustainable entrepreneurship is becoming popular, different from the traditional profit-based entrepreneurship (Muñoz et al., 2018; Terán-Yépez et al., 2020; Kummitha and Kummitha, 2021). This is because it builds a business that balances the triple bottom line’s economic, social, and environmental aspects (Belz and Binder, 2017). Students act as the agents of change to become sustainable entrepreneurs (Ploum et al., 2017). Therefore, it is essential to study the factors influencing students to become environmentally conscious entrepreneurs.

Previous studies focused on the entrepreneurial intentions (Jena, 2020; Sharahiley, 2020; Vamvaka et al., 2020), and only a few investigated the intentions of becoming a sustainable green entrepreneur (Middermann et al., 2020) and become a green entrepreneur (Wang and Peng, 2020). This study proposed a holistic model to determine the factors encouraging students to become environmentally conscious entrepreneurs. Moreover, the theory of planned behavior (TPB) (Ajzen, 1991), the Hofstede’s model of national cultures (Hofstede, 2011), and cognition knowledge from the Schraw’s Metacognitive Theory (Schraw and Dennison, 1994; Schraw and Moshman, 1995) were used to explore the factors driving green entrepreneurial intentions. It determined the role of contextual factors as a moderating effect, using an entrepreneurial support model (ESM) by Turker and Sonmez Selcuk (2009).

### Theory of Planned Behavior

Nascent business is influenced by the supporting psychological traits, behavior, and the founder (Davidsson and Honig, 2003; Backes-Gellner and Moog, 2013). A previous study explained that TPB could conceptualize creating a new business through intentionality (Maheshwari, 2021). This theory states that intention describes one’s disposition to be involved in a certain behavior and directly determines such behavior. The TPB approach predicts entrepreneurial intention (Krueger et al., 2000; Engle et al., 2008; Liñán and Chen, 2009; Naushad, 2018), utilized in previous entrepreneurship studies to explain and predict behavior due to its vast applicability (Henley et al., 2017; Dao et al., 2021; Haddad et al., 2021; Maheshwari, 2021). It hypothesizes that entrepreneurial intentions can be predicted by motivation and its three independent constructs, including attitude toward behavior, subjective norm, and perceived behavioral control.

*Attitude toward behavior* (ATB) shows one’s evaluation degree or judgment of behavior, where more likable behavior increases their positive perception of starting a business (Ajzen, 1991). Measurement of attitude toward a behavior is based on one’s opinion (Ajzen, 1991). The more positive the attitude toward behavior, the stronger the entrepreneurial intention (Fayolle and Gailly, 2015; Zapkau et al., 2015; Fietze and Boyd, 2017).

*Subjective norms* (SNs) indicate the perceived social pressures when deciding whether to perform a certain behavior. They are based on the conviction of whether a significant individual or group of reference approves or disapproves of an individual’s decision to start a business and its importance to that individual (Ajzen, 1991). More subjective norms supporting the individual’s effort to become an entrepreneur strengthen their entrepreneurial intentions (Fayolle and Gailly, 2015; Fietze and Boyd, 2017; Esfandiar et al., 2019).

*Perceived behavioral control* (PBC) is the perceived ease or difficulty of performing a certain behavior (Ajzen, 1991). Positive entrepreneurial self-efficacy increases an individual’s entrepreneurial intentions to start a business (Fietze and Boyd, 2017; Esfandiar et al., 2019; Nowiński et al., 2019; Schmutzler et al., 2019). In contrast, perceived controllability is an individual’s perception of having sufficient control over the required resources to manage a challenge successfully (Ajzen, 2002). The more the positive perception of sufficient control over the required resources to manage a challenge, the stronger an individual’s intentions to pursue an entrepreneurial career (Fayolle and Gailly, 2015; Tounés et al., 2018). The following hypotheses were formulated based on the literature review:

H1. ATB positively and significantly affects green entrepreneurial intentions.

H2. SNs positively and significantly affects green entrepreneurial intentions.

H3. PBC positively and significantly affects green entrepreneurial intentions.

#### Cultural Values

This study’s cultural values adopted five dimensions from the Hofstede’s model of national cultures (Hofstede, 2011). (1) Power distance shows the diverse arrangements of the essential human inequality issue. (2) Uncertainty avoidance shows the society’s stress level in confronting an obscure future. (3) Individualism vs. collectivism shows the people’s integration into primary groups. (4) Masculinity vs. femininity shows the division of women and men’s emotional parts. (5) Long-term vs. short-term orientation shows the option of center for the people’s endeavors, including the future or the present and the past. Singelis and Brown (2006) believed that an individual’s attitudes, values, and self-concept are shaped by culture, which, when measured at the individual level, can improve the understanding of the relationship between culture and individual behavior.

The Hofstede’s cultural values approach represents a brief taxonomy of significant cultural dimensions, accounting for individuals’ behavioral preferences within a certain society. This approach views a continuous application in the cross-cultural entrepreneurship studies, such as the effects of gender inequalities (Santos et al., 2016; Shinnar et al., 2017; Smith et al., 2019), values, customs, and codes of conduct (Weiss et al., 2019), the level of uncertainty avoidance in a society (Fietze and Boyd, 2017), collectivism (Walter and Block, 2016) and individualism values (Liñán et al., 2015; Roman and Maxim, 2017), and long- and short-term orientations (Bogatyreva et al., 2019) on entrepreneurial intentions. The following hypothesis was formulated based on the literature review:

H4. Cultural values positively and significantly affect green entrepreneurial intentions.

#### Knowledge of Cognition

Knowledge of cognition denotes the reflective measure of metacognition and an individual’s awareness of the components influencing the structures of knowledge and learning (Schraw and Dennison, 1994; Schraw and Moshman, 1995). Previous studies discovered the crucial roles of knowledge development (McKelvie et al., 2017), exchange (Zhang et al., 2016), and structures (Loi et al., 2016) in understanding behavior to start a business. Despite the rarity of studies discussing cognitive knowledge’s role in forming an entrepreneurial intention, this study understood that the Indonesian culture finds the knowledge of cognition necessary for growing entrepreneurial intentions. Therefore, it is necessary to discuss the individual’s understanding of their or the general cognition (Schraw and Moshman, 1995; Barbosa et al., 2007; Sánchez, 2012) on entrepreneurial intentions.

The cognitive approach uses entrepreneurs’ cognitive aspects to learn and explain their behavior on identifying opportunities for creating and developing businesses. “Cognitive style” characterizes certain ways of processing information on entrepreneurship behavior. Cognitive literature distinguishes two streams, cognitive structure, and studies on the cognitive process (Sánchez et al., 2011). The social cognitive theory introduces the knowledge structure aspect, a mental model (cognition) used to achieve personal effectivity in certain situations. Therefore, since entrepreneurship consists of individuals or teams that create products or services, cognitive psychology is increasingly used to establish phenomena on entrepreneurship (Sánchez, 2011).

The knowledge of cognition used the three aspects of metacognitive awareness by Schraw and Moshman (1995), including declarative, procedural, and conditional knowledge. Declarative knowledge is about oneself as a learner and factors influencing their performance. Procedural knowledge describes the execution of procedural skills, while the conditional knowledge when one knows when and why to apply various cognitive actions. Greater declarative, procedural, and conditional knowledge strengthens the entrepreneurial intentions (Bagheri and Lope Pihie, 2013; Pihie et al., 2013). The following hypothesis was proposed based on the literature review:

H5. Knowledge of cognition positively and significantly affects green entrepreneurial intentions.

#### Contextual Factors

A few studies explored the roles of contextual factors in fostering entrepreneurial intentions, such as institutional and an entrepreneur’s family or non-work context (Shepherd et al., 2018). This study’s contextual factors denoted the ESM by Turker and Sonmez Selcuk (2009), advising that entrepreneurial intentions could be a work of structural, educational, and relational support. Universities’ support helps form the students’ entrepreneurial intentions (Lüthje and Franke, 2003; Saeed et al., 2015). Families, universities, and economic institutions positively foster entrepreneurial intentions in the students and youth (Arrighetti et al., 2016; Fietze and Boyd, 2017; Pérez-Macías et al., 2019).

Previous studies showed the effects of organizational and regional contexts on the students’ propensity to start a business (Bergmann et al., 2016). For example, the effects of universities and the environment on entrepreneurial intentions (Davey et al., 2016; Shirokova et al., 2017; Feola et al., 2019), and the effects of economic and family on perceived desirability and feasibility of business students (Henley et al., 2017). Positive family and university contexts support the attitude toward entrepreneurship, subjective norms, and PBC into entrepreneurial intentions (Fietze and Boyd, 2017). Furthermore, family, economic institutions, and university support influence the youth or students’ entrepreneurial intentions (Arrighetti et al., 2016; Pérez-Macías et al., 2019). The University’s support influences students’ entrepreneurial intentions through entrepreneurial self-efficacy (Lüthje and Franke, 2003; Saeed et al., 2015). However, there lacked studies on contextual factors as reinforcers or inhibitors of the relationship between entrepreneurial intentions and its antecedents. Other studies regarded the contextual factors’ role as moderators of the relationship between entrepreneurial self-efficacy (Schmutzler et al., 2019), attitude, subjective norms, and behavioral control (Arranz et al., 2019) to determine entrepreneurial intentions. Few studies identified contextual factors as a moderator variable hence this study comprehensively included contextual factors and tested them as a moderator variable.

Cole (2007) examined cultural factors influencing entrepreneurship development in Indonesia, using Hofstede’s cultural dimensions to investigate the relationship between cultural dimensions and entrepreneurship. The results showed that high power distance, uncertainty avoidance, and collectivism inhibited entrepreneurship. Some studies asserted that lack of knowledge and structural support hinders entrepreneurship and that the government can impede it on a greater scale (Cole, 2007).

Knowledge of cognition includes metacognition, a high-level cognitive process that organizes what one knows and recognizes self, tasks, situations, and the environment to support effective and adaptive cognitive functioning. The metacognitive activity consists of self-consciousness, thinking and reflection, strategic thinking, planning, considering plans, knowing what is known, and overseeing oneself. This sets a foundation for an entrepreneurial mindset (Haynie and Shepherd, 2009; Haynie et al., 2010). The following hypotheses were formulated based on the literature review:

H6a. Contextual factors toward green entrepreneurial intentions moderate the relationship between attitude toward behavior and green entrepreneurial intention.

H6b. Contextual factors toward green entrepreneurial intentions moderate the relationship between subjective norms and green entrepreneurial intention.

H6c. Contextual factors toward green entrepreneurial intentions moderate the relationship between perceived behavioral control and green entrepreneurial intention.

H6d. Contextual factors toward green entrepreneurial intentions moderate the relationship between cultural values and green entrepreneurial intention.

H6e. Contextual factors toward green entrepreneurial intentions moderate the relationship between knowledge of cognition and green entrepreneurial intention.

H7. Contextual factors positively and significantly affect green entrepreneurial intentions.

#### Green Entrepreneurial Intentions

Entrepreneurial intentions are essential in an entrepreneurship process as the first fundamental step in business creation (Molino et al., 2018). Several models and theories have been developed in the last 20 years to explain entrepreneurial intentions (Molino et al., 2018), including the TPB (Ajzen, 1991), “implementing entrepreneurial ideas” (Bird, 1988), Shapero’s entrepreneurial event (Shapero and Sokol, 1982), and the recent Luthje and Franke’s model (Lüthje and Franke, 2003). The Shapero’s entrepreneurial event model describes three crucial factors for entrepreneurial intentions, perceived desirability, feasibility, and propensity to act. Perceived desirability is a strong attractiveness toward a business venture, while feasibility indicates people’s confidence about creating a business. Finally, the propensity to act concerns the disposition to act based on opportunities (Shapero and Sokol, 1982).

Krueger (1993) and Krueger and Brazeal (1994) supported Shapero’s findings which stated that attitude is linked to entrepreneurial intentions, especially perceived desirability, and feasibility (Shapero and Sokol, 1982). Krueger (1993) established that early entrepreneurial exposure affected intentions through perceived feasibility. Positive past encounters influenced perceived desirability to start a business (Krueger and Carsrud, 1993). Furthermore, the intention-based models derived from TPB suggested that entrepreneurial intentions function the perceived feasibility and desirability of an entrepreneurial act (Krueger et al., 2000).

This study used TPB as a model to measure entrepreneurial intentions. Several previous studies measured entrepreneurial intention as a construct, including a TPB (van Gelderen et al., 2008; Varamäki et al., 2015; Zapkau et al., 2015; Arranz et al., 2019; Pérez-Macías et al., 2019; Weiss et al., 2019) or Shapero’s entrepreneurial event model stream (Henley et al., 2017; Vuorio Anna et al., 2018; Esfandiar et al., 2019). However, this study argued that measuring entrepreneurial intentions as a whole construct could not distinguish entrepreneurial intentionalities, explicit or unambiguous. Therefore, gauging entrepreneurial intentions should explore individuals with explicit and unambiguous entrepreneurial intentionalities, such as those with real intentions or who opted for self-employment (Franco et al., 2010).

Green entrepreneurship is intentional, planned behavior, and a complex process involving various stages (Yi, 2021). It is a business that combines environmental awareness with entrepreneurial action, changing toward a sustainable business model (Schaper, 2002; Gibbs and O’Neill, 2014). Previous studies showed that the relationship between entrepreneurship, the environment, and sustainable development includes various thoughts of schools. It is presented in different terms such as ecological (Schaper, 2002; Linnanen, 2016; Gast et al., 2017), environmental (Dean and McMullen, 2007; Corbett and Montgomery, 2017), green (Pihie et al., 2013; Demirel et al., 2019; Ramayah et al., 2019; Yi, 2021), and sustainable entrepreneurship (Jayaratne et al., 2019; Westman et al., 2019; Sargani et al., 2020).

Franco et al. (2010) categorized entrepreneurial intentions into three spheres, including non-founders as students who do not want to be self-employed, potential founders who do not exclude the possibility, and founders who intend to be self-employed, who have begun first activities toward that, or already self-employed. This study excluded the non-founders and focused on the respondents who desired entrepreneurship and started a business.

## Methodology

### Research Design

**FIGURE 1:**
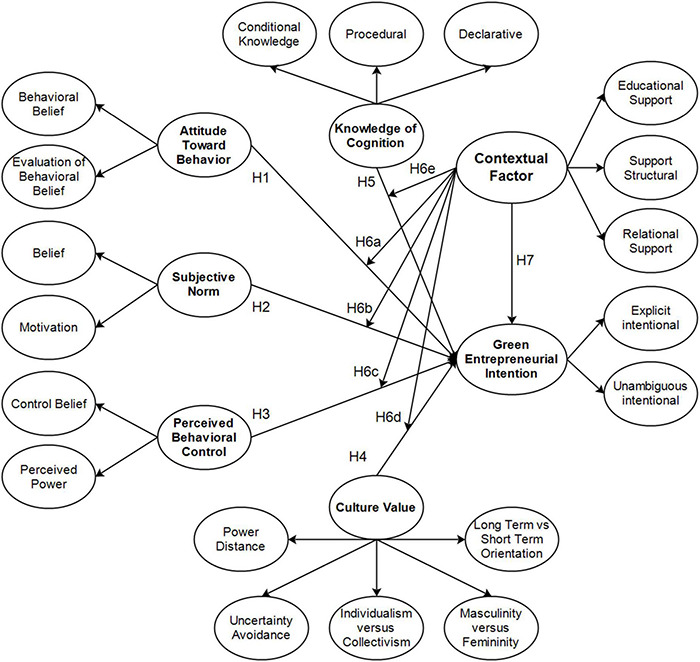
The research model.

This study advanced management science, focusing on the TPB, cultural values, knowledge of cognition, entrepreneurial intentions, and contextual factors as mediators (Figure 1). The model was developed by adopting previous relevant studies analyzing students at private universities in Jakarta adopting entrepreneurship-based curricula. This study is categorized as applied research, exploring theories and concepts (Zikmund et al., 2013) and generating a model for investigating the students’ green entrepreneurial intentions. It is verificative, testing theories or previous findings to produce results that consolidate or diminish them.

### Population and Sample

The population of this research is students from 10 private universities in Jakarta. They come from three faculties: mathematics and natural sciences (FMIPA), engineering, and social sciences (management, accounting, and communication). The total population is unknown with certainty, and the sample size uses the Lemeshow formula with a 5% margin of error and 50% outcome prevalence (Lemeshow et al., 1990). The results obtained 385 samples and were selected using purposive and snowball techniques (Anderson et al., 2019), only students who intend to become entrepreneurs. The questionnaire data collection starts from November 2020 to February 2021 using the Google form. As a result, from a target of 385 samples, only 305 questionnaires were returned and deserved to be analyzed so that the success rate in data collection was 79.22% (Hendra and Hill, 2018).

### Measurement

Each construct’s measurements were developed from previous studies, with adjustments based on the research problem (Table 1). The attitude toward behavior had two subconstructs measured by 5 questions, while the subjective norm construct had two subconstructs. The PBC had two subconstructs, while 6 questions measured the subjective norm constructs and PBC. Furthermore, culture value had five subconstructs measured by 20 questions. The knowledge of cognition had three subconstructs measured by 16 questions. Finally, green entrepreneurial intentions had two subconstructs measured by 5 questions. The research model consisted of moderating contextual factors with three subconstructs measured by 8 questions. A five-point Likert scale (1 = strongly disagree and 5 = strongly agree) was applied to measure all the questions.

**TABLE 1:** Measurement of constructs.

Constructs	Sub-constructs	Item	Researchers
Attitude toward behavior	Behavioral belief	1.Being an entrepreneur provides me with more benefits than losses. 2.Given a chance, I would start a green business. 3.Given the resources, I would start a green business.	Harris Michael and Gibson Shanan, 2008; Vinogradov et al., 2013; Yurtkoru et al., 2014; Fayolle and Gailly, 2015
	Evaluation of behavioral belief	1.Being an entrepreneur provides me with more profits than losses. 2.Given the opportunity, I would start a business.	
Subjective norm	Belief	1.My family members think that I should pursue a green entrepreneurial career. 2.My closest friends think that I should pursue a green entrepreneurial career. 3.People important to me think I should pursue a green entrepreneurial career.	Turker and Sonmez Selcuk, 2009; Iakovleva et al., 2011; Raza et al., 2018; Tounés et al., 2018
	Motivation	1.I care about what my family members think when I pursue an entrepreneurial career. 2.I care about what my closest friends think when I pursue an entrepreneurial career. 3.I care about what talented people think when I pursue an entrepreneurial career.	
Perceived behavioral control	Control belief	1.When I start a business, I will have a high probability of succeeding. 2.Starting a business will be easy for me.	Millman et al., 2010; Yurtkoru et al., 2014; Raza et al., 2018
	Perceived power	1.I am ready to start a decent business. 2.I can control creating a business. 3.I know the required practical details to start a business. 4.I can develop an entrepreneurial project.	
Culture values	Power distance	1.I have enough time for personal or family life. 2.I have good physical learning conditions (ventilation, lighting, and adequate classrooms). 3.I have a wonderful relationship with my lecturer. 4.I am studying at the campus.	Hofstede, 2011
	Uncertainty avoidance	1.Do you feel nervous in class? 2.Are you afraid to express disagreements with lecturers? 3.I can trust most people. 4.One can be a good lecturer without answering students’ questions well on things that can improve their understanding.	
	Individualism vs. collectivism	1.I work well with other students. 2.The lecturer consults the students before deciding on the learning process. 3.I have a higher score. 4.There is learning variation (diversity) and adventure elements at the campus.	
	Masculinity vs. femininity	1.Personal stability and stability. 2.Austerity. 3.Persistence (perseverance). 4.Respect tradition.	
	Long term vs. short term orientation	1.Having two lecturers in the same subject, I should avoid them. 2.Competition between students is dangerous. 3.I should not break the university rules, not only when students think it is in the university’s best interest. 4.It is someone’s fault for failing in life.	
Knowledge of cognition	Declarative	1.I understand my intellectual qualities and weaknesses. 2.I know the vital information to learn. 3.I am great at organizing information. 4.I know what the lecturer expects me to learn. 5.I am great at remembering information. 6.I have control over how well I learn. 7.I am great at judging how well I understand something. 8.I learn more when I am inquisitive about the subject.	Schraw and Moshman, 1995; Bagheri and Lope Pihie, 2013; Pihie et al., 2013
	Procedural	1.I attempt to use strategies that have worked in the past. 2.I have a particular reason for each strategy I use. 3.I am mindful of the studying strategies I use. 4.I discover myself using helpful learning strategies.	
	Conditional knowledge	1.I use distinctive learning strategies depending on the circumstance. 2.I can motivate myself to learn when I need to. 3.I use my intellectual strengths to compensate for my weaknesses. 4.I know when each strategy is the most viable.	
Green entrepreneurial Intention	Explicit intentional.	1.I think I will start a business. 2.I considered founding my business. 3.Given the opportunity, I would prefer to operate my own business.	van Gelderen et al., 2008
	Unambiguous intentional.	1.Considering your actual situation, I would operate my own business. 2.I will run my own firm in the next 5 years.	Franco et al., 2010
Contextual factors	Educational support	1.The university education energizes me to develop creative entrepreneurial ideas. 2.The university creates my entrepreneurial aptitudes and capacities. 3.The university provides essential knowledge on entrepreneurship.	Turker and Sonmez Selcuk, 2009
	Support structural	1.The Indonesian economy provides various opportunities for entrepreneurs. 2.In Indonesia, entrepreneurs are empowered by an underlying including private systems, public, and non-governmental organizations. 3.Taking bank loans is difficult for entrepreneurs in Indonesia.	
	Relational support	1.My close network (work, school, and neighborhood) would support me if I were an entrepreneur. 2.My family members would support me if I were an entrepreneur.	

The primary data were measured using Partial Least Square-Structural Equation Modeling (PLS-SEM). The use of PLS-SEM with SmartPLS version 3.3.3 can examine the measurement model either formatively, reflectively, or both together. In addition, it is easy to measure constructs with a hierarchy, and high-statistical power even though the distribution of the data does not meet the normality assumption. The research data does not meet the assumption of normality and hierarchical construct measurements (Hair et al., 2019), so PLS-SEM is the best choice for data analysis. PLS-SEM comprising two models, the measurement and structural (Hair et al., 2019). The measurement model measures the validity and reliability, while the structural estimates the path between the constructs (Hair et al., 2017). Higher-order models were used to test the validity because each construct was multidimensional or contained several levels, for example, translating the construct into several factors (Crocetta et al., 2021). Finally, the reflective model was used for each measurement.

## Data Analysis and Results

### Student Characteristics

The first stage of the questionnaire validity process involved filtering the respondents’ answers. The inclusion was the respondents who answered *yes* to intending to become an entrepreneur or have started a business. The next stage analyzed the respondents’ characteristics as supporting information for the findings (Table 2).

**TABLE 2:** Cross-tabulation of student characteristics.

	Intend to become an entrepreneur		Have started a business		
	*n*	%	*n*	%	
Gender	Male	147	67.4%	39	44.8%
	Female	71	32.6%	48	55.2%
Disciplines	Math	92	42.2%	11	12.6%
	Technical	84	38.5%	19	21.8%
	Social science	42	19.3%	57	65.5%
College year	1st years	45	20.6%	17	19.5%
	2nd years	43	19.7%	32	36.8%
	3rd years	70	32.1%	21	24.1%
	Final years	60	27.5%	17	19.5%
Family members who become green entrepreneurs	Father	85	39.0%	20	23.0%
	Mother	47	21.6%	12	13.8%
	Brother or sister	43	19.7%	37	42.5%
	Grand-Father	0	0.0%	12	13.8%
	Grand-Mother	15	6.9%	6	6.9%
	Others	28	12.8%	0	0.0%

Table 2 shows that 65.5% of Social Science students owned their businesses, while 67.4% of male students intended to become entrepreneurs. Those owning their business were 36.8% of 2nd students and 42.5% had started their business because they had a brother or sister as an entrepreneur. All the students needed support from the universities to provide startup business simulations and seminars or training from successful entrepreneurs. The results concluded that the universities held a paramount role in building the students’ entrepreneurial intentions.

### Measurement Model

The results of the reflective measurement model were evaluated through confirmatory factor analysis (CFA) to test the latent construct’s validity and reliability (Hair et al., 2019). The measurement model test consisted of convergent and discriminant validity and reliability tests. Convergent validity shows the principle that the manifest variables should be highly correlated, assessed by the rule of thumb that the loading value > 0.7 and the average variance extracted (AVE) value > 0.5 (Hair et al., 2017). Furthermore, discriminant validity used the Fornell–Larcker criterion method to compare the AVE root and correlation value between latent variables (Hair et al., 2017). The reliability test used composite reliability (CR) to test the instrument’s accuracy, consistency, and measuring constructs. It is assessed by the rule of thumb that the CR value must be above 0.7 (Hair et al., 2017).

#### Convergent Validity and Composite Reliability

Table 3 presents the convergent validity testing with higher-order models. In the first-order model, all the items had a loading factor value > 0.7, similar to the second-order for each subconstruct. The AVE at the sub construct level had a value above 0.5, meaning that the convergent validity test was satisfactory. Furthermore, reliability testing for the subconstruct showed a CR value > 0.7, proving that the latent variables were valid and reliable as a measuring instrument for each construct.

**TABLE 3:** Convergent validity, average variance extracted (AVE), and composite reliability.

Constructs	Sub-constructs	Second order (SLF)	Item	First order (SLF)	Composite reliability	AVE
Attitude toward behavior	Behavioral belief	0.952	BB1	0.884	0.844	0.645
			BB2	0.791		
			BB3	0.726		
	Evaluation of behavioral belief	0.896	EBB1	0.820	0.798	0.664
			EBB2	0.810		
Subjective norm	Belief	0.922	BE1	0.868	0.882	0.715
			BE2	0.815		
			BE3	0.852		
	Motivation	0.921	Mot1	0.844	0.885	0.720
			Mot2	0.831		
			Mot3	0.870		
Perceived behavioral control	Control belief	0.844	CB1	0.904	0.874	0.776
			CB2	0.858		
	Perceived power	0.958	PP1	0.807	0.907	0.710
			PP2	0.869		
			PP3	0.835		
			PP4	0.858		
Culture values	Power distance	0.867	PD1	0.863	0.918	0.737
			PD2	0.872		
			PD3	0.830		
			PD4	0.869		
	Uncertainty avoidance	0.894	UA1	0.880	0.883	0.656
			UA2	0.874		
			UA3	0.687		
			UA4	0.783		
	Individualism vs. Collectivism	0.862	IC1	0.820	0.877	0.641
			IC2	0.761		
			IC3	0.841		
			IC4	0.778		
	Masculinity vs. Femininity	0.797	MF1	0.810	0.877	0.640
			MF2	0.743		
			MF3	0.845		
			MF4	0.798		
	Long term vs. Short term orientation	0.878	LTST1	0.796	0.856	0.598
			LTST2	0.727		
			LTST3	0.783		
			LTST4	0.786		
Knowledge of cognition	Conditional knowledge	0.730	CK1	0.832	0.861	0.609
			CK2	0.708		
			CK3	0.748		
			CK4	0.827		
	Procedural	0.838	PRO1	0.772	0.873	0.632
			PRO2	0.801		
			PRO3	0.832		
			PRO4	0.773		
	Declarative	0.928	DECL1	0.713	0.904	0.542
			DECL2	0.793		
			DECL3	0.767		
			DECL4	0.770		
			DECL5	0.769		
			DECL6	0.722		
			DECL7	0.697		
			DECL8	0.648		
Green entrepreneurial intentions	Explicit intentional	0.917	EI1	0.804	0.820	0.604
			EI2	0.827		
			EI3	0.695		
	Unambiguous intentional	0.798	UI1	0.811	0.771	0.627
			UI2	0.772		
Contextual factors	Educational support	0.607	ES1	0.923	0.942	0.844
			ES2	0.920		
			ES3	0.913		
	Support structural	0.851	SS1	0.878	0.899	0.749
			SS2	0.892		
			SS3	0.825		
	Relational support	0.761	RS1	0.914	0.879	0.784
			RS2	0.856		

#### Discriminant Validity

The discriminant validity represents a construct’s empirical difference from others or the extent to be measured. The Fornell and Larcker criterion is one of the methods for assessing discriminant validity. It postulates that a latent variable should share more variance with its indicators than other latent variables. Furthermore, the results suggested that the model had a good discriminant validity (Fornell and Larcker, 1981; Table 4).

**TABLE 4:** Discriminant validity—Fornell–Larcker criterion.

	BB	BE	CB	CK	DECL	EBB	EI	ES	IC	LTST	MF	Mot	PD	PP	PRO	RS	SS	UA	UI
BB	**0.803**																		
BE	0.426	**0.845**																	
CB	0.469	0.421	**0.881**																
CK	0.630	0.298	0.243	**0.780**															
DECL	0.778	0.373	0.362	0.523	**0.736**														
EBB	0.718	0.412	0.382	0.652	0.713	**0.815**													
EI	0.543	0.341	0.374	0.457	0.467	0.553	**0.777**												
ES	0.429	0.237	0.238	0.330	0.377	0.446	0.469	**0.919**											
IC	0.374	0.288	0.201	0.333	0.395	0.413	0.384	0.295	**0.800**										
LTST	0.360	0.193	0.201	0.275	0.377	0.389	0.355	0.242	0.695	**0.793**									
MF	0.341	0.165	0.202	0.215	0.379	0.351	0.312	0.222	0.559	0.784	**0.800**								
Mot	0.428	0.699	0.445	0.273	0.413	0.453	0.311	0.261	0.307	0.190	0.142	**0.848**							
PD	0.358	0.207	0.165	0.304	0.297	0.363	0.397	0.204	0.717	0.633	0.540	0.157	**0.859**						
PP	0.402	0.375	0.655	0.189	0.308	0.351	0.322	0.156	0.176	0.174	0.196	0.364	0.117	**0.843**					
PRO	0.628	0.421	0.339	0.484	0.666	0.662	0.497	0.443	0.327	0.263	0.237	0.451	0.280	0.276	**0.795**				
RS	0.462	0.291	0.293	0.385	0.358	0.500	0.420	0.186	0.278	0.255	0.251	0.296	0.265	0.304	0.382	**0.885**			
SS	0.495	0.324	0.315	0.404	0.344	0.517	0.497	0.213	0.254	0.199	0.148	0.253	0.344	0.305	0.394	0.583	**0.865**		
UA	0.372	0.265	0.193	0.321	0.370	0.408	0.384	0.218	0.728	0.702	0.601	0.235	0.783	0.128	0.324	0.266	0.337	**0.810**	
UI	0.519	0.378	0.290	0.312	0.551	0.550	0.493	0.288	0.263	0.234	0.250	0.319	0.269	0.355	0.525	0.495	0.512	0.291	**0.792**

*BB, Behavioral Belief; BE, Belief; CB, Control Belief; CK, Conditional Knowledge; DECL, Declarative; EBB, Evaluation of Behavioral Belief; EI, Explicit intentional; ES, Educational Support; IC, Individualism vs. Collectivism; LTST, Long Term vs. Short Term Orientation; MF, Masculinity vs. Femininity; PD, Power Distance; PP, Perceived Power; PRO, Procedural; RS, Relational Support; SS, Support Structural; UA, Uncertainty Avoidance; UI, Unambiguous intentional.*

*Bold values indicates that discriminant validity has been met. Example: the correlation between the BB construct and its own construct is 0.803, which is greater than the other constructs in column BB.*

### Structural Model

**FIGURE 2:**
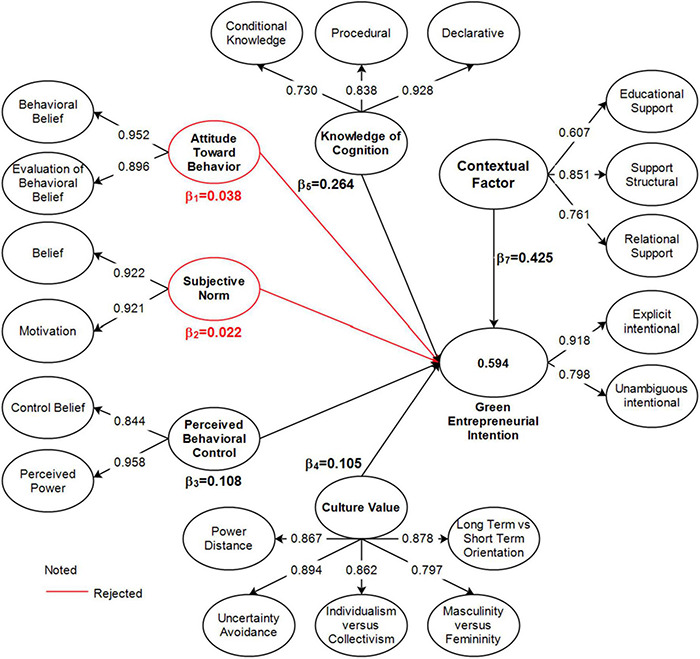
Structural model before moderation.

Structural model testing examines the effects of exogenous variables on endogenous (Hair et al., 2017). This study tested 6 hypotheses in the structural model summarized in Figure 2 and Table 5. Attitudes toward behavior and subjective norms showed no direct and significant effect on green entrepreneurial intentions (β_1_ = 0.038; *t*_1_ = 0.394; *p*_1_ = 0.694; β_2_ = 0.022; *t*_2_ = 0.464; *p*_2_ = 0.643). In contrast, PBC, cultural values, knowledge of cognition, and contextual factors showed a direct and significant effect on green entrepreneurial intentions (β_3_ = 0.038; *t*_3_ = 0.394; *p*_3_ = 0.694; β_4_ = 0.022; *t*_4_ = 0.464; *p*_4_ = 0.643; β_5_ = 0.022; *t*_5_ = 0.464; *p*_5_ = 0.643; β_6_ = 0.022; *t*_6_ = 0.464; *p*_6_ = 0.643). This supported the direct effect hypothesis (H3–H6), while proving that the direct hypothesis (H1 and H2) had no significant effect. In addition, contextual factors were the most significant predictor of green entrepreneurial intentions followed by knowledge of cognition, PBC, and cultural values. Better support from the university, government, and mates strengthened the entrepreneurial intentions.

**TABLE 5:** Hypotheses testing (bootstrapping 500 samples).

	Path	Std (β)	*T*-values	*P*-values	Decision	R-square	F-Square
H1.	ATB→GEI	0.038	0.394	0.694	Rejected	0.594	0.001
H2.	SN→ GEI	0.022	0.464	0.643	Rejected		0.001
H3.	PBC→ GEI	0.108	2.320	0.021	Accepted		0.020
H4.	CV→GEI	0.105	2.246	0.025	Accepted		0.021
H5.	KC→ GEI	0.264	3.037	0.003	Accepted		0.040
H6.	CF→GEI	0.425	7.372	0.000	Accepted		0.231

*ATB, Attitude Toward Behavior; SN, Subjective Norms; PBC, Perceived Behavioral Control; CV, Cultural Values; KC, Knowledge of Cognition; CF, Contextual Factors; GEI, Green Entrepreneurial Intentions.*

The structural model results explained that attitudes toward behavior, subjective norms, PBC, cultural values, and cognitive knowledge contributed 59.4% in shaping students’ intentions to become green entrepreneurs.

#### Predictive Relevance of the Model

The blindfolding procedure was one of the criteria for evaluating the quality of the model. *Q*^2^ predictive relevance measured how well the model’s observed values were generated and the estimated parameters (Hair et al., 2017). A *Q*^2^-value above 0 indicated that the model had predictive relevance, while below 0 showed a lack of predictive relevance. Table 6 presents the model’s predictive relevance and obtained a *Q*^2^-value of 0.269 > 0 or 26.9%, supporting its predictive quality.

**TABLE 6:** Predictive relevance (blindfolding method).

Total	SSO	SSE	*Q*^2^ (= 1-SSE/SSO)
Green entrepreneurial intentions	1.525.000	1.114.651	0.269

#### Assessment of the Effect Size

Effect size (*f*^2^) is a quantitative measure of exogenous variables’ effect on the endogenous variables based on changes in R-Square (Cohen, 2013). The *f*^2^-value interpretation by Cohen (2013) was 0.02, showing a small effect, where 0.15 and 0.35 show a moderate and a large impact on the structural level, respectively (Hair et al., 2019). Table 5 showed the effect size results, where all relationships had a small effect except between contextual factors and green entrepreneurial intentions with a moderate impact of f^2^ = 0.231).

### Moderating Effect

**FIGURE 3:**
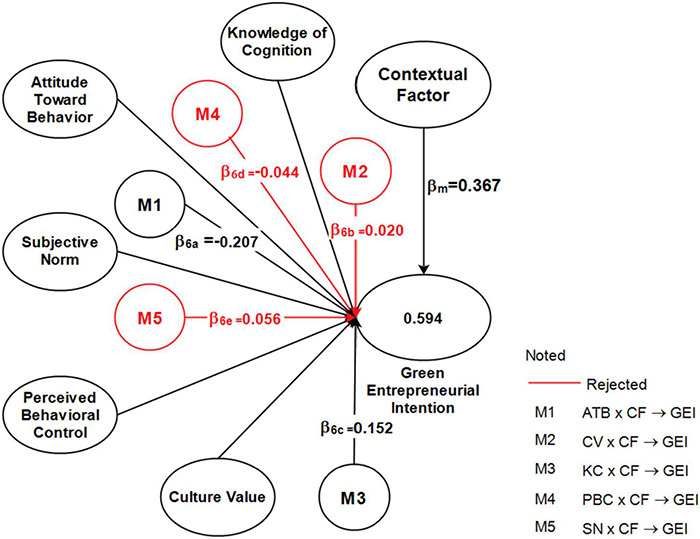
Structural model—moderating effect.

Figure 3 and Table 7 shows that the moderating effect of contextual factors on the relationship between cultural values, PBC, and subjective norms with green entrepreneurial intentions was not significant (β_*m*2_ = 0.020; *t*_*m*2_ = 0.583; *p*_*m*2_ = 0.560; β_*m*4_ = –0.044; *t*_*m*4_ = 0.943; *p*_*m*4_ = 0.346; β_*m*5_ = 0.056; *t*_*m*5_ = 1.256; _*m*5_ = 0.210). On the other hand, contextual factors significantly moderated the relationship between attitudes toward behavior and knowledge of cognition with green entrepreneurial intentions (β_*m*1_ = –0.207; *t*_*m*1_ = 2.711; *p*_*m*1_ = 0.007; β_*m*3_ = 0.152; *t*_*m*3_ = 2.020; *p*_*m*3_ = 0.044). This rejected the moderating hypotheses (H6b, H6d, and H6e) and accepted the moderating hypotheses H6a and H6c.

**TABLE 7:** Moderating effect.

Hypotheses	Code	Path	Std (β)	*T*-values	*P*-values	Decision
H6a	M1	ATB × CF → GEI	–0.207	2.711	0.007	Accepted
H6b	M2	CV × CF → GEI	0.020	0.583	0.560	Rejected
H6c	M3	KC × CF → GEI	0.152	2.020	0.044	Accepted
H6d	M4	PBC × CF → GEI	–0.044	0.943	0.346	Rejected
H6e	M5	SN × CF → GEI	0.056	1.256	0.210	Rejected

**FIGURE 4:**
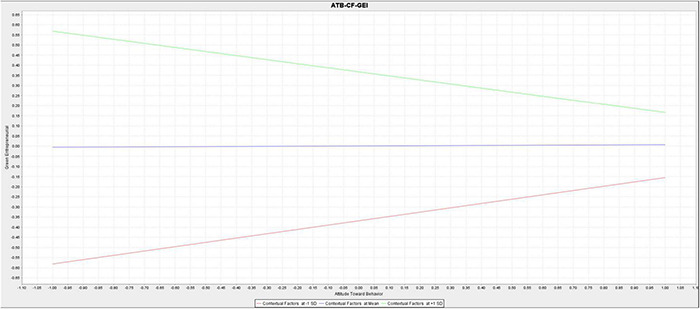
The simple slope for the moderating effect of contextual factors on the relationship between attitudes toward behavior and entrepreneurial intentions.

**FIGURE 5:**
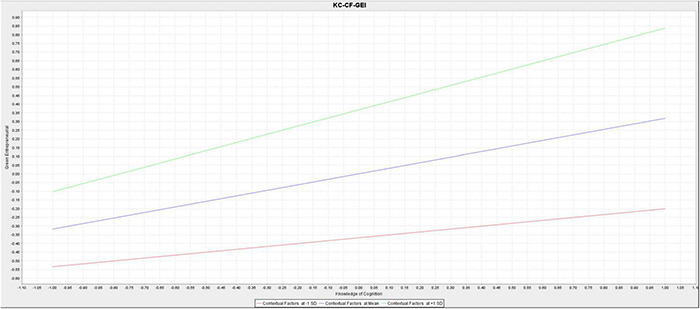
The simple slope for the moderating effect of contextual factors on the relationship between knowledge of cognition and entrepreneurial intentions.

Figures 4, 5 show the size of the moderating effect, indicating that the direct effect of attitudes toward behavior on green entrepreneurial intentions was insignificant. However, when moderated by the contextual factors, the effect becomes significant with a negative coefficient. This indicates that contextual factors can directly change the direction of the relationship between attitudes toward behavior and green entrepreneurial intentions in a negative and significant direction. Therefore, contextual factors significantly decrease the effect of attitudes toward behavior on green entrepreneurial intentions. Knowledge of cognition positively and significantly impacts green entrepreneurial intentions, becoming stronger when moderated by the contextual factors moderate. Therefore, contextual factors significantly strengthen the effects of knowledge of cognition on green entrepreneurial intentions.

The sample included students owning a business and working while studying. Students accepted entrepreneurship courses in only one semester, which is insufficient to comprehensively analyze the entrepreneurship stages until company formation or launch and growth. Therefore, contextual factors such as educational, structural, and relational support do not impact students’ positive business behavior. Students receive the knowledge and skills to generate ideas, evaluate opportunities, and plan from lecturers based on the cognitive knowledge. They know that the external and internal factors that can support them set up a business during the learning process. Therefore, the contextual factors such as educational, structural, and relational support strengthen their cognitive knowledge to start businesses.

## Discussion, Limitations, And Future Research

### Discussion

This study focused on understanding the students’ behaviors in Jakarta, Indonesia, on pursuing green entrepreneurship after graduation through TPB factors (attitude toward behavior, subjective norms, and PBC), cultural, and knowledge of cognition. It investigated the role of contextual factors as a mediator of TPB. The model’s results without moderation proved that PBC, cultural values, and knowledge of cognition influenced the students’ green entrepreneurial intentions. These results are in line with Tkachev and Kolvereid (1999), Krueger et al. (2000), Autio et al. (2001), and Gird and Bagraim (2008), which investigated the students’ entrepreneurial intentions. They explained that attitude toward behavior, subjective norms, and PBC positively affected green entrepreneurial intentions. Autio et al. (2001) explained that PBC is the most prominent in studying entrepreneurial intentions. However, this study showed that the subjective norms and attitude toward behavior did not give significantly influence. This finding was common in the previous studies, such as Krueger et al. (2000), Alam et al. (2019), and Majeed et al. (2021), which confirmed that all the TPB factors could predict entrepreneurial intentions except subjective norms and attitude toward behavior ruled out in the regression analysis despite showing a significant correlation. Autio et al. (2001) and Al-Jubari (2019) identified that subjective norms negatively influenced entrepreneurial intentions among the postgraduate business students. The results showed that the role of PBC when starting a business should be more sensitive and measured. It was the weakest predictor of green entrepreneurial intentions; hence, universities should build students’ attitudes toward becoming entrepreneurs through lecturers than only presenting case studies on the entrepreneurship.

The results showed that the cultural values had a significant positive effect on green entrepreneurial intentions. Zahra and George (2002) established that entrepreneurship measures are supported by beliefs and values in the social environment (culture), appreciating or inhibiting behaviors such as innovation, creativity, and risk-taking. Karayiannis (1993) reported that the entrepreneurs form a belief attitude through cultural heritage and life experiences from other people. Before starting a business, they follow certain people’s paths in their immediate surroundings, especially, their family background. As earlier stated, the sampled students run their own or intend to start businesses with family support.

Knowledge of cognition successfully predicted the understanding of the students’ green entrepreneurial intentions. This is in line with Barbosa et al. (2007) and Sánchez (2012) inspiring the study on the role of cognition knowledge among the students. Cognition is a new factor that influences entrepreneurial intention, hence, this study contributed that the students’ metacognitive consciousness can be improved through education or classroom training (Barbosa et al., 2007; Sánchez, 2012). Therefore, the teachers should know cognition to understand the students’ strengths and weaknesses in the learning process, especially green entrepreneurship. They can allow the students to develop their knowledge and insights through successful entrepreneurs’ social interactions and experiences. Finally, better students’ cognition knowledge strengthens their intentions to become green entrepreneurs.

Contextual factors were examined as a moderator of attitude toward behavior, subjective norms, PBC, cultural values, and knowledge of cognition to green entrepreneurial intentions. The results showed that these factors, including educational, structural, and relational support, could not predict students’ green entrepreneurial intentions fully. However, constructively, contextual factors can moderate the relationship between attitudes toward behavior, subjective norms, PBC, cultural values, and cognitive knowledge with green entrepreneurial intentions. Several researchers examined contextual factors as direct predictors of entrepreneurial intentions. For example, Hassan et al. (2021) found that government policy support and financial access as contextual factors did not affect the students’ motivation to become entrepreneurs. Tran Anh and Von Korflesch (2016) designed a contextual factors model as a predictor to cultivate entrepreneurial intentions. Meanwhile, Lukman et al. (2021) best tested the role of universities as a moderator to foster social entrepreneurial intentions, and the results had a positive and significant effect. Because of the lack of researchers who analyze contextual factors (educational, structural, and relational support) as moderation, the results of this study provide new insights in assessing green entrepreneurial intentions among the students.

The practical implications, universities must cultivate students’ green entrepreneurial intentions. They can encourage lecturers to teach entrepreneurship courses to share creative ideas, case studies, and in-depth insights into the business world. Furthermore, based on the structural support, the Government should facilitate the students’ green entrepreneurial intentions through economic stability, incubation training, private sector support, and loan interest reduction for the entrepreneurs. The Government and universities must synergize to develop incubator institutions evenly to realize innovative young entrepreneurs. In addition, the results found that the support from the students’ immediate environment affected their entrepreneurial intentions, including close friends or family. Family members are role models for the students to become entrepreneurs. For example, parents teach entrepreneurship lessons by inviting their children to be involved in business, help solve problems and think creatively to advance the business. Therefore, the role of parents has a significant impact on extending the intention of green entrepreneurship.

Finally, this study concludes that PBC, culture value, knowledge of cognition, and contextual factors directly affect green entrepreneurial intentions. At the same time, attitude toward behavior and the subjective norm has no effect. Simultaneously, contextual factors succeeded in moderating the relationship between attitudes toward behavior, subjective norms, PBC, cultural values, and cognitive knowledge with green entrepreneurial intentions. Partially, contextual factors have strengthened the relationship between knowledge of cognition and green entrepreneurial intentions and weakened the relationship between attitude toward behavior and green entrepreneurial intentions.

### Limitations and Future Research

The measurement focused on the students’ entrepreneurial intentions, disregarding their actions to become entrepreneurs after graduating. However, the sample covered those intending to start and those running their businesses. Second, the sample size was small at 305; hence, the future studies should employ a longitudinal model involving the students’ university years to run their businesses after graduation. This study suggested using contextual factors as a predictor to investigate green entrepreneurial intentions and expand the respondents’ coverage to understand the students’ intentions comprehensively. In addition, the sample should include private and public universities to observe the different behaviors of students.

## Data Availability Statement

The original contributions presented in the study are included in the article/supplementary material, further inquiries can be directed to the corresponding author/s.

## Author Contributions

HP, RI, and YY contributed to the conception and design of the research and wrote part of the script. HP and YY conducted a literature review and conceptual model. RI carried out statistical analysis and wrote the first draft of the manuscript. All authors contributed to manuscript revision, reading, and approving submitted versions.

## Conflict of Interest

The authors declare that the research was conducted in the absence of any commercial or financial relationships that could be construed as a potential conflict of interest.

## Publisher’s Note

All claims expressed in this article are solely those of the authors and do not necessarily represent those of their affiliated organizations, or those of the publisher, the editors and the reviewers. Any product that may be evaluated in this article, or claim that may be made by its manufacturer, is not guaranteed or endorsed by the publisher.
